# Structural Distortion in MnO_2_ Nanosheets and Its Suppression by Cobalt Substitution

**DOI:** 10.3390/nano7100295

**Published:** 2017-09-25

**Authors:** Shinya Suzuki, Masaru Miyayama

**Affiliations:** Department of Applied Chemistry, School of Engineering, The University of Tokyo, 7-3-1 Hongo, Bunkyo-ku, Tokyo 113-8656, Japan; miyayama@fmat.t.u-tokyo.ac.jp

**Keywords:** MnO_2_, Co–Mn oxide, nanosheets, optical absorption, X-ray absorption spectroscopy, local structure

## Abstract

Co–Mn oxide nanosheets with the chemical composition H_0.23_Co_0.23_Mn_0.77_O_2_ (C23M77NS) and MnO_2_ nanosheets (M100NS) were prepared by exfoliation of layer-structured oxides via chemical processing in an aqueous medium. The optical properties of C23M77NS and M100NS were compared using UV-Vis spectroscopy, and the valence states of Mn and Co and local structures around them were examined using X-ray absorption spectroscopy. M100NS with an average Mn valence of 3.6 exhibits large structural distortion, whereas C23M77NS with an average Mn valence of 4.0 does not exhibit structural distortion. Spontaneous oxidization of Mn occurs during ion-exchange and/or exfoliation into nanosheets. These results have originated the hypothesis that structural distortion determines the valence state of Mn in compounds with CdI_2_-type-structured MnO_2_ layers.

## 1. Introduction

Atomic-layered materials have recently attracted significant attention because they exhibit a variety of anomalous properties that are distinct from those of three-dimensional bulk systems. Since graphene was first isolated from graphite in 2004 [1], many studies have reported on its unusual electronic and physical properties, such as high electron mobility, Dirac-cone states [2] and quantum Hall effects [3]. While graphene has potential applications as a conductor, various nanosheets based on non-graphene materials, with a wide variety of applications, have also been synthesized. For instance, considering only oxide nanosheets, semiconducting [4,5], dielectric [6], electron-conducting [7,8], ion-conducting [9,10], ferromagnetic [11,12], photocatalytic [13,14] and redoxable nanosheets [5,7,8] have been obtained. In addition, chalcogenide nanosheets, such as piezoelectric [15] or ferromagnetic [16] MoS_2_, have also been widely studied. 

Adding functionalities to the above nanosheets is mainly carried out by elemental substitution, often with the help of computational chemistry. For example, Cheng et al. have reported that two-dimensional diluted magnetic semiconductors based on MoS_2_ nanosheets can be obtained by doping with transition metals [17], while Wang et al. predicted that oxide nanosheets with half-metallic electronic structures can be prepared by introducing oxygen vacancies to MnO_2_ nanosheets with semiconductor-like electronic structures [18]. 

MnO_2_ nanosheets exhibit various functionalities and have been studied as electrodes of lithium-ion secondary batteries [5], those of supercapacitors [19], catalyst [20], biosensing materials [21], and so on [22]. MnO_2_ nanosheets have been reported to have a hexagonal CdI_2_-type structure [23], and density functional theory (DFT) calculations have been conducted using such a CdI_2_-type structure as the initial structure [18]. We recently reported that Mn–Ni oxide nanosheets have a distorted crystal structure due to the cooperative Jahn–Teller effect of Mn^3+^ [24], which suggests the possibility that MnO_2_ nanosheets also exhibit a distorted crystal structure. The results of DFT calculations are greatly affected by the crystal structure used, since fundamental information on the structures of these systems is important for rationalizing and eventually predicting their physical properties. In the present work, we therefore compared the structural properties of MnO_2_ and Co–Mn oxide nanosheets in detail using X-ray absorption spectroscopy (XAS).

## 2. Results

**Figure 1:**
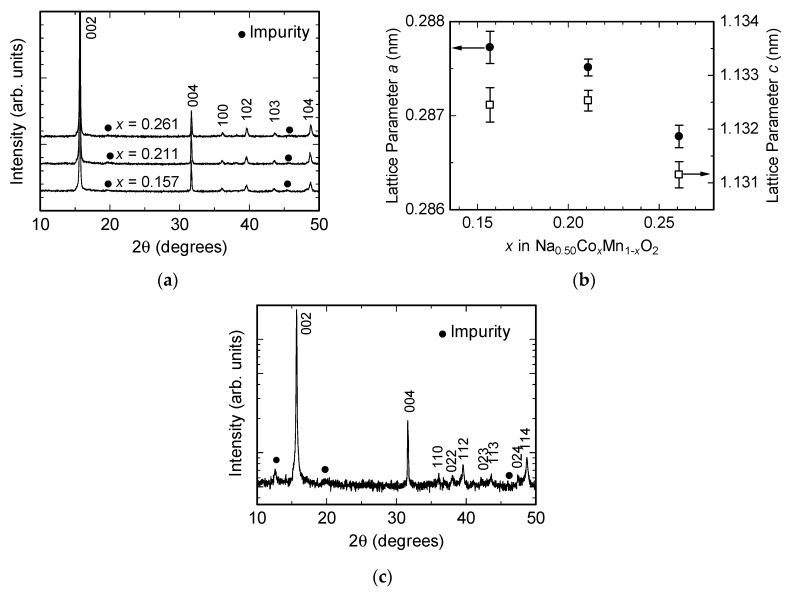

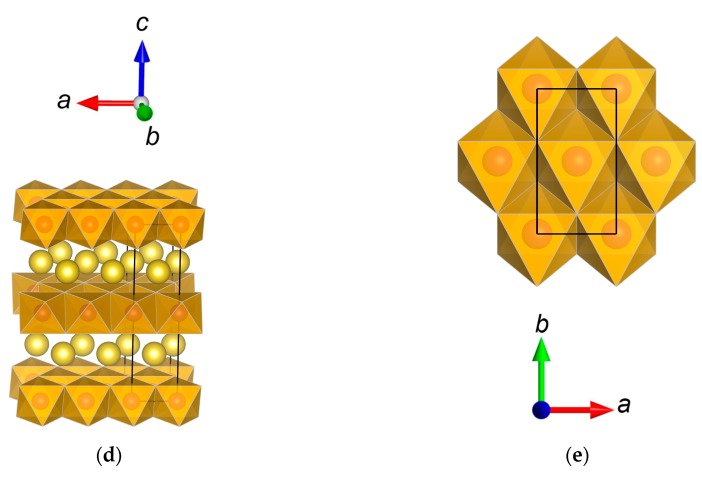
(**a**) X-ray diffraction (XRD) patterns of the prepared Na_0.50_(Co*_x_*Mn_1−*x*_)O_2_ (*x* = 0.157, 0.211 or 0.261) systems; (**b**) Rietveld-refined lattice parameters of Na_0.50_(Co*_x_*Mn_1−*x*_)O_2_ as a function of Co content *x*; (**c**) XRD pattern of prepared Na_0.50_MnO_2_; (**d**) model of the crystal structure of Na_0.50_MnO_2_; (**e**) top view of the oxide layer of (**d**). The black solid lines in (**d**,**e**) indicate the unit cell.

Figure 1a shows the powder X-ray diffraction (XRD) patterns of the prepared oxide materials, with a nominal composition of Na_0.50_(Co*_x_*Mn_1−*x*_)O_2_ (*x* = 0.15, 0.20 or 0.25); the actual chemical compositions were determined as Na_0.551_(Co_0.157_Mn_0.843_)O_2_, Na_0.520_(Co_0.211_Mn_0.789_)O_2_ and Na_0.508_(Co_0.261_Mn_0.739_)O_2_. All XRD peaks were indexed to a hexagonal P2-type layered structure (space group *P*6_3_/*mmc*) with the only exceptions a few weak peaks originating from impurities. The lattice parameters were refined by Rietveld analysis using the RIETAN-FP code [25]. The powder XRD and Rietveld refinement results are shown in Figure 1a,b, respectively. The lattice parameter *a* decreased almost linearly with an increase in Co content *x*, which can be interpreted in terms of the smaller size of Co^3+^ (whose ionic radius in the low-spin, hexacoordinated state is 54.5 pm [26]) compared to that of Mn^3+^ (whose high-spin state radius is 64.5 pm [26]). This result thus confirms that Co substitution was successful. The lattice parameter *c*, on the other hand, did not change linearly with the Co content, possibly as a result of the strong influence of the interlayer Na content on this parameter. Figure 1c shows the XRD patterns of Na_0.50_MnO_2_, in which all peaks can be indexed to the orthorhombic P2-type layered structure (space group *Cmcm*), except for a few weak peaks associated with impurities. Despite the known challenges associated with the preparation of pure Na_0.50_MnO_2_, the oxide obtained here was almost pure. Figure 1d,e shows models of the crystal structure of Na_0.50_MnO_2_. Rietveld refinement of the lattice parameters yielded *a* = 0.2832 nm, *b* = 0.5203 nm and *c* = 1.1303 nm, corresponding to a lattice orthorhombicity (defined as *b*/√3*a*) of 1.06. 

**Figure 2:**
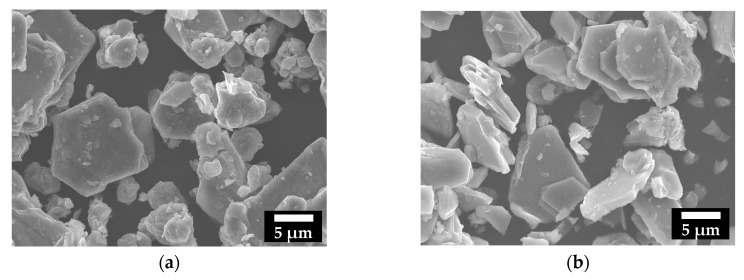
Scanning electron microscopy (SEM) images of (**a**) Na_0.508_Co_0.261_Mn_0.739_O_2_ and (**b**) Na_0.50_MnO_2_.

Figure 2a,b shows scanning electron microscopy (SEM) images of prepared Na_0.508_(Co_0.261_Mn_0.739_)O_2_ and Na_0.50_MnO_2_ powders, revealing plate-like particles in the size range of 2–10 μm. 

Na_0.508_(Co_0.261_Mn_0.739_)O_2_ powders were reacted with nitric acid to form the proton-exchanged form of the Na–Co–Mn oxide. A greenish-brown dispersion of Co–Mn oxide nanosheets was obtained, with a yield >60%, by exfoliation of the proton-exchanged Na–Co–Mn oxide. The chemical composition of these Co–Mn oxide nanosheets, determined by inductively-coupled plasma atomic emission spectroscopy (ICP-AES) and XAS, was H_0.23_Co_0.23_Mn_0.77_O_2_·*n*H_2_O, indicating that the Co/Mn ratio had changed upon exfoliation. Since the XRD analysis discussed above indicated small amounts of impurities in the Na–Co–Mn oxides, centrifugal separation was used to remove them from the nanosheets. Therefore, the Co/Mn ratio measured for the Co–Mn oxide nanosheets is the actual one. The obtained nanosheet dispersion is referred to as C23M77NS hereafter. Similar to the above, an orange dispersion of MnO_2_ nanosheets (hereafter M100NS) was also prepared from Na_0.50_MnO_2_, with a yield of approximately 30%. 

**Figure 3:**
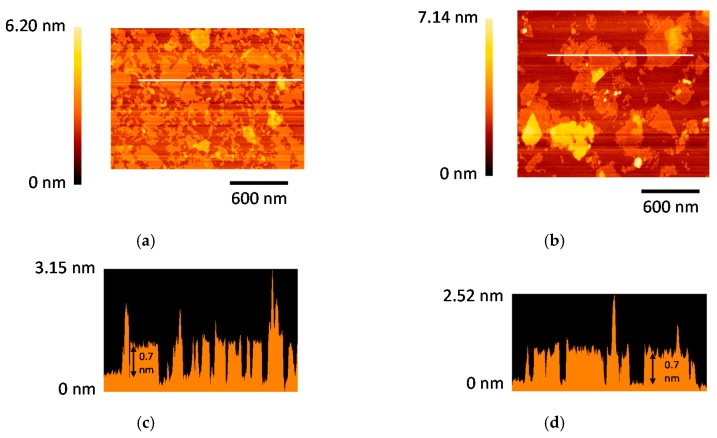
Atomic force microscopy (AFM) images of (**a**) H_0.23_Co_0.23_Mn_0.77_O_2_ nanosheets (C23M77NS) and (**b**) MnO_2_ nanosheets (M100NS) deposited onto mica substrates. Panels (**c**) and (**d**) show cross-sectional profiles of the regions indicated by the white lines in Panels (**a**) and (**b**), respectively.

Figure 3a,b shows atomic force microscopy (AFM) images of C23M77NS and M100NS deposited on mica substrates; small flakelets with lateral dimensions of 50–500 nm can be observed. Figure 3c,d shows the cross-sectional AFM profiles corresponding to the regions indicated by the white lines in Figure 3a,b respectively. The structures of the nanosheets are clearly visible in these figures, and nanosheets of a 0.7-nm thickness (one oxide layer [27]) were observed for both C23M77NS and M100NS. Monolayer nanosheets of Co–Mn oxide and MnO_2_ were thus successfully obtained. 

**Figure 4:**
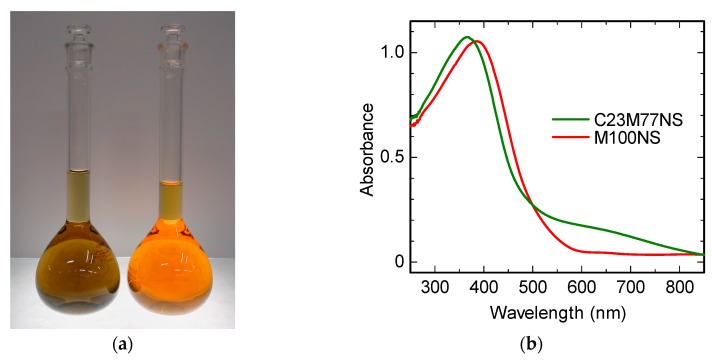
(**a**) Photographs of the colloidal suspensions of C23M77NS (left) and M100NS (right); (**b**) UV-visible optical absorption spectra of C23M77NS and M100NS. The concentration of the dispersion was 0.1 mmol (Mn + Co) dm^−3^, and the cell path length was 1 cm.

Digital photographs of the C23M77NS and M100NS dispersions are shown in Figure 4a, whereas Figure 4b shows the corresponding UV-Vis absorption spectra. C23M77NS and M100NS exhibited broad absorption peaks at 366 and 386 nm, respectively. Omomo et al. previously reported similar optical properties, with an absorption peak centered around 374 nm for MnO_2_ nanosheets prepared by exfoliation of layer-structured H_0.13_MnO_2_ [5]. Replotting the UV-Vis spectra in Tauc form allowed estimation of the direct allowed band gaps of C23M77NS and M100NS as 2.75 and 2.62 eV, respectively. We recently reported a direct allowed band gap of 2.66 eV for H_0.46_Mn_0.81_Ni_0.19_O_2_ nanosheets in which the valence state of Mn is 3.9 [24]. The observed absorption bands here are due to the d-d transition of Mn^4+^, and any difference in the corresponding peak wavelength would be mainly caused by structural distortion of the MnO_6_ octahedra, as further discussed below. The C23M77NS sample also showed a shoulder peak around 600 nm, which can be attributed to the d-d transition of Co^3+^.

**Figure 5:**
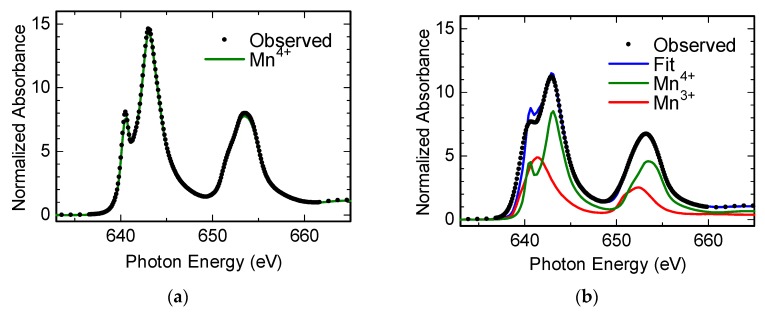
X-ray absorption near-edge structure (XANES) spectra measured in total electron yield mode at the Mn L_2,3_-edge of restacked (**a**) C23M77NS and (**b**) M100NS.

C23M77NS and M100NS were restacked by reacting them with a LiOH aqueous solution, and the restacked products were also subjected to XAS measurements. Specifically, the valence states of Mn and Co were determined by XAS measurements at the L_2,3_-edge. Figure 5a,b shows X-ray absorption near-edge structure (XANES) spectra measured in total electron yield mode at the Mn L_2,3_-edge of restacked C23M77NS and M100NS, respectively. The average valence values for Mn in restacked C23M77NS and M100NS were 4.0 and 3.6, respectively, whereas the valence of Co in restacked C23M77NS, also determined by XAS, was 3.0 (results not shown).

**Figure 6:**
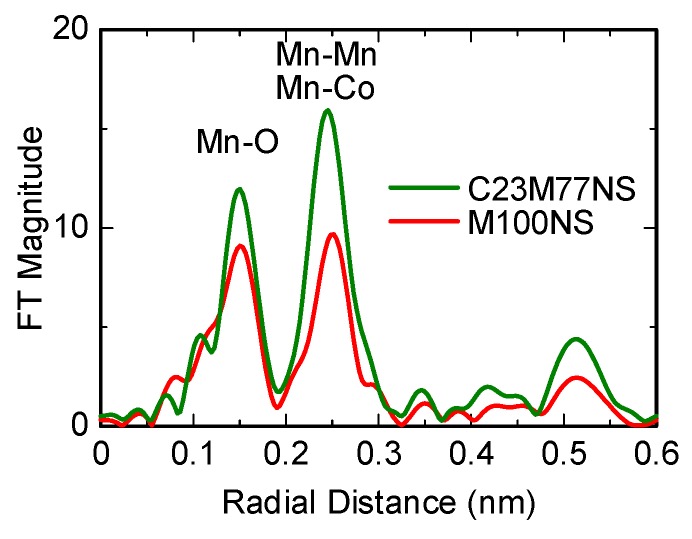
Fourier transforms (FTs) of the Mn K-edge extended X-ray absorption fine structure (EXAFS) spectra of restacked C23M77NS and M100NS.

The local Mn structure in both the restacked C23M77NS and M100NS was studied by extended X-ray absorption fine structure (EXAFS) analysis. The Fourier transforms (FTs) of the Mn K-edge EXAFS spectra of restacked C23M77NS and M100NS are shown in Figure 6. The first peak around 0.15 nm is due to the Mn-O contacts, whereas the one around 0.25 nm corresponds to Mn-Mn or Mn-Co interactions. The areas and intensities of these two main peaks were smaller for restacked M100NS than for C23M77NS, showing that the structural distortion of MnO_6_ units is larger in M100NS than in C23M77NS. The Fourier-transformed EXAFS spectra previously recorded for MnO_2_ nanosheets obtained from K_0.45_MnO_2_ [23,28] were almost identical to those recorded in this study for the nanosheets obtained from Na_0.50_MnO_2_, which suggests that the crystal structure of MnO_2_ nanosheets remains the same, regardless of the starting material used for their synthesis. In the previous studies above, MnO_2_ nanosheets were considered to have hexagonal CdI_2_-type structures without distortion. Here, we have shown for the first time that MnO_6_ units are distorted in MnO_2_ nanosheets.

The local structure around Mn atoms in C23M77NS and M100NS was determined under the following two assumptions: (1) MnO_6_ units in C23M77NS are not distorted; (2) MnO_6_ distortion in M100NS is of the same type as that present in the starting Na_0.5_MnO_2_ material with the orthorhombic P2-type layered structure (space group *Cmcm*). The first assumption is certainly reasonable, since C23M77NS is free from Jahn–Teller Mn^3+^ ions. Mn–O and Mn–Mn distances were determined by nonlinear curve-fitting analysis of the inverse FT to *k* space with a two-shell model composed of six-fold coordination for both Mn–O and Mn–Mn [23,28]. The structural parameters obtained by fitting of the EXAFS spectra are summarized in Table 1; the estimated Mn–O and Mn–Mn distances of 0.1899 and 0.2908 nm, respectively, are roughly consistent with previously reported values for MnO_2_ nanosheets, which were also determined under the assumption of six-fold coordination for both Mn-O and Mn-Mn [23,28]. In order to assess the validity of the local structure, the oxidation numbers of the Mn ions in C23M77NS and M100NS were numerically evaluated by bond valence sum (BVS) analysis [29], in which the effective valence is calculated as:

BVS = Σ exp{(R_0_ − R)/0.037} (1)

where *R*_0_ is the bond valence parameter for Mn^4+^ (0.1753 nm) derived from Brown’s table [29], and *R* is the Mn–O interatomic distance listed in Table 1. The calculated BVS values for Mn in C23M77NS and M100NS are 4.0 ± 0.1 and 3.3 ± 0.4, respectively, estimates that are in good agreement with the observed oxidation number for Mn shown in Figure 5; thus, the second assumption discussed above is also reasonable. The calculated orthorhombicity parameter *R*(Mn–Mn(1))/*R*(Mn–Mn(2)) of 1.19 was significantly larger than that of the starting Na_0.5_MnO_2_, showing that exfoliation into nanosheets promoted structural distortion.

## 3. Discussion

The optical absorption of M100NS showed a red shift compared to that of C23M77NS, which may be ascribed to the split of both the unoccupied *e*_g_ and occupied *t*_2g_ levels caused by the reduction in symmetry of MnO_6_ octahedra due to the cooperative Jahn–Teller effect of Mn^3+^ contained in M100NS. The Co substitution into MnO_2_ nanosheets resulted in the disappearance of the structural distortion. 

The nominal Mn valences were 3.50 and 3.67 for the starting Na_0.50_MnO_2_ and Na_0.508_(Co_0.261_Mn_0.739_)O_2_, respectively. Thus, Mn is spontaneously oxidized during ion-exchange and/or exfoliation. This spontaneous oxidation/reduction of Mn has also been observed during the preparation of MnO_2_ nanosheets from K_0.45_MnO_2_ [23]; Mn is oxidized during ion-exchange of the interlayer cations and reduced by exfoliation into nanosheets. The cause of oxidation is not clear, but the results above suggest that structural distortion determines the valence state of Mn for CdI_2_-type-structured MnO_2_ layers. That is: (1) Mn is spontaneously oxidized during ion-exchange of interlayer cations from Na^+^ or K^+^ to H_3_O^+^, since MnO_6_ octahedra are stable in their slightly distorted state in the H_3_O^+^-exchanged form of Na_0.50_MnO_2_ or K_0.45_MnO_2_; (2) MnO_6_ octahedra are stable in their regular state, especially in the H_3_O^+^-exchanged form of Na_0.508_(Co_0.261_Mn_0.739_)O_2_, due to the effect of substituted Co^3+^, and Mn is spontaneously oxidized to Mn^4+^ (free from Mn^3+^ Jahn–Teller ions) to enable this; and (3) MnO_6_ octahedra in Mn100NS are stable in their largely distorted state and Mn^4+^ is spontaneously reduced partially to Mn^3+^ Jahn–Teller ions. If the above hypothesis is true, then CdI_2_-type-structured Co–Mn oxide nanosheets with rather small Co contents would contain Mn^3+^ and Co^2+^ Jahn–Teller ions.

## 4. Materials and Methods

Layer-structured Na–Co–Mn and Na–Mn oxides were synthesized by a conventional solid-state reaction. Reagent-grade Na_2_CO_3_, Mn_2_O_3_, MnO_2_ and Co_3_O_4_ were mixed and ball-milled in acetone. Each dried mixture was pressed to form pellets, which were then heated at 1100 °C for 20 h. A 5% excess of Na_2_CO_3_ was used to compensate for the loss due to volatilization upon heating. The resulting pellets were ground and used as starting powder materials. The XRD measurements were conducted using a D8 Advance diffractometer (Bruker AXS, Karlsruhe, Germany) equipped with a VANTEC-1 position-sensitive detector (Bruker AXS, Karlsruhe, Germany) and a Cu target X-ray tube. The SEM measurements were performed using a JSM-7000F microscope (JEOL, Tokyo, Japan).

The proton-exchanged form of the Na–Mn oxide was prepared by reacting the powder form of the oxide with 1 M HNO_3_ at room temperature for 5 d in order to allow ion exchange, with the HNO_3_ solution replaced daily. MnO_2_ nanosheets were then prepared by exfoliating the proton-exchanged Na–Mn oxide by reacting with a tetrabutylammonium hydroxide (TBAOH) aqueous solution (TBAOH/Na–Mn oxide molar ratio = 2:1) for 10 d at room temperature. After 10 d, the unreacted particles were separated by centrifugation at 5000 rpm, and a colloidal dispersion of nanosheets was obtained as the supernatant. The same procedure as above was used to prepare Co–Mn oxide nanosheets from Na–Co–Mn oxide. The chemical compositions of the different nanosheet types were determined by ICP-AES using a SPS3100 analyzer (Hitachi High-Tech Science, Tokyo, Japan). The size and shape of each nanosheet type were determined by AFM, using a Nanocute/NanoNavi-II instrument (Hitachi High-Technologies, Tokyo, Japan). The optical absorption properties of the nanosheets were examined using a V-570 spectrophotometer (JASCO, Tokyo, Japan). 

The prepared MnO_2_ and Co–Mn oxide nanosheets were restacked by reacting them with 1 M LiOH aqueous solution, which was followed by washing with purified water and drying at room temperature. XAS measurements of the restacked nanosheets were conducted at both Mn K- and L_2,3_-edges. The K-edge measurements were conducted using a transmission method at the BL-12C beamline of the Photon Factory of the High Energy Accelerator Research Organization (Tsukuba, Japan), whereas the L_2,3_-edge ones were conducted in total electron yield mode at the BL-11 beamline of the Synchrotron Radiation (SR) Center of Ritsumeikan University (Kusatsu, Japan). The FTs of the Mn K-edge XAS spectra were obtained with *k*^3^ weighting in a *k* range of 2.8–13.2 Å^−1^. The structural parameters were determined by curve-fitting procedures using the Athena-Artemis software [30]. The models of the crystal structures were prepared using VESTA software [31]. 

## 5. Conclusions

Mn and Co–Mn oxide nanosheets were prepared by aqueous exfoliation from Na_0.50_MnO_2_ and Na_0.508_(Co_0.261_Mn_0.739_)O_2_, respectively. Extended X-ray absorption fine structure analysis at the Mn K-edge revealed that MnO_2_ nanosheets (M100NS) exhibit structural distortion due to the Jahn–Teller effect associated with Mn^3+^ ions. The structural distortion in M100NS was suppressed by Co substitution. H_0.23_Co_0.23_Mn_0.77_O_2_ and M100NS showed optical absorption peaks at 366 and 386 nm, respectively; the difference is mainly due to the structural distortion in M100NS. The structural information obtained in this study will contribute to achieve a better understanding of the physical properties of nanosheets, which will in turn allow the exploration of novel applications of these systems.

## Figures and Tables

**Table 1:** Structural parameters of restacked C23M100NS and M100NS determined by analyzing the Mn K-edge EXAFS spectra. CN, *R* and *σ* denote coordination number, bond length and Debye–Waller factor, respectively.

Interaction	Mn-O (1)			Mn-O (2)			Mn-Mn (1)			Mn-Mn (2)		
	CN	*R* (nm)	*σ* (nm)	CN	*R* (nm)	*σ* (nm)	CN	*R* (nm)	*σ* (nm)	CN	*R* (nm)	*σ* (nm)
C23M77NS	6	0.1904	0.0048	-	-	-	6	0.2862	0.0051	-	-	-
M100NS	4	0.1899	0.0051	2	0.2215	0.0200	4	0.2908	0.0061	2	0.2449	0.0121
